# Three-Dimensional Imaging and Gene Expression Analysis Upon Enzymatic Isolation of the Tongue Epithelium

**DOI:** 10.3389/fphys.2020.00825

**Published:** 2020-07-21

**Authors:** Christian T. Meisel, Pierfrancesco Pagella, Cristina Porcheri, Thimios A. Mitsiadis

**Affiliations:** Orofacial Development and Regeneration, Centre for Dental Medicine, Institute of Oral Biology, University of Zurich, Zurich, Switzerland

**Keywords:** tongue epithelium, epithelial stem cells, three-dimensional imaging, keratin14, cell proliferation, Sox2, tissue organization, enzymatic separation

## Abstract

The tongue is a complex organ involved in a variety of functions such as mastication, speech, and taste sensory function. Enzymatic digestion techniques have been developed to allow the dissociation of the epithelium from the connective tissue of the tongue. However, it is not clear if the integrity and three-dimensional architecture of the isolated epithelium is preserved, and, furthermore if this tissue separation technique excludes its contamination from the mesenchymal tissue. Here, we first describe in detail the methodology of tongue epithelium isolation, and thereafter we analyzed the multicellular compartmentalization of the epithelium by three-dimensional fluorescent imaging and quantitative real-time PCR. Molecular characterization at both protein and transcript levels confirmed the exclusive expression of epithelial markers in the isolated epithelial compartment of the tongue. Confocal imaging analysis revealed that the integrity of the epithelium was not affected, even in the basal layer, where areas of active cell proliferations were detected. Therefore, the preservation of both the architecture and the molecular signature of the tongue epithelium upon enzymatic tissue separation enable further cellular, molecular and imaging studies on the physiology, pathology, and regeneration of the tongue.

## Introduction

The tongue is a complex organ located on the floor of the mouth whose muscular core is protected by a multi-layered epithelial mucosa. While the ventral part of the tongue is lined by a thin non-keratinized epithelium, its dorsal part is covered by a specialized, stratified, highly keratinized mucosa that consists of four types of papillae (Takahashi et al., 2019). Filiform papillae cover the entire rough surface of the tongue’s surface, fungiform papillae are located at the anterior 2/3 of the tongue, and foliate and circumvallate papillae are linked to the taste buds that transmit the umami, bitter, salty, sour, and sweet taste (Nguyen et al., 2012; Barlow, 2015; Snyder and Bartoshuk, 2016; Mistretta and Kumari, 2017). The basal layer of the oral epithelium and the underlying mesenchyme are separated by a basement membrane called the lamina propria (Squier and Brogden, 2011; Castillo-Azofeifa et al., 2018). Epithelial cells are juxtaposed to each other and exhibit specific structural features according to their differentiation status. Epithelial stem cells residing in this layer undergo asymmetric divisions to self-renew and give rise to transit amplifying progenitors (Okubo et al., 2009; Tanaka et al., 2013). The generated undifferentiated progenitors translocate to the next epithelial layer and, after progressing through the amplification stage, start to differentiate and progressively acquire elongated cell shapes (Watt, 1989; Castillo-Azofeifa et al., 2018). To date, the study of the epithelial stem cells of the tongue has been hampered by the lack of efficient tools for the visualization and manipulation of the deeper epithelial layers, such as the basal layer.

The epithelium and mesenchyme of the tongue are tightly adherent to each other and several enzymatic separation techniques have been proposed and performed for the isolation of the epithelium (Ookura et al., 2002; Ozdener et al., 2006; Venkatesan et al., 2016). However, it is important to conserve the structural integrity of the isolated tissue and avoid any possible mesenchymal tissue inclusion. Tissue impurity can largely modify the read-out of the analyses and produce misleading results. As a consequence, some of the previous genetic and structural analyses have been based on mixed tissues that contained both components (Tang et al., 2015; Sur et al., 2018). To avoid these issues, we initially tested the structural integrity of the enzymatically isolated tongue epithelium by immunofluorescence and three-dimensional (3D) imaging, and thereafter confirmed its purity by gene expression analysis. Advanced confocal imaging can then be applied for the 3D visualization of the tissue, which can be studied in its integrity. Upon enzymatic digestion, the organization of the epithelial multilayer is accessible and can be observed at cellular and subcellular level. We here provide the characterization of the basal layer using specific markers for progenitor cells to identify their distribution within the tissue. Exploiting this system, the physiological organization of the stem cell niche can be visualized with minimal alteration of their native architecture. Our findings could be helpful for studies focusing on the analysis of specific epithelial cell populations in tongue physiology, regeneration and pathology (e.g., cancers).

## Materials and Methods

### Animals

The mice were maintained and handled according to the Swiss Animal Welfare Law (Animal Welfare Act of 16 December 2005). This study was approved by the Cantonal Veterinary office, Zurich (License 197/2017). C57/BL6J and *Sox2*^CreER^; *R26*^mT/mG^ transgenic mice were used for this study. *Sox2*^CreER^; *R26*^mT/mG^ transgenic mice were generated by crossing *R26*^mT/mG^ (*Gt(ROSA)26Sor^*tm4(ACTB–tdTomato,–EGFP)Luo*^*; Jax: 007576) mice with *Sox2*^CreER^ (B6;129S-Sox2<tm1(cre/ERT2)Hoch>/J(#017593); MGI: 177655) mice. The double-fluorescent Cre reporter *R26*^mT/mG^ mice constitutively express membrane-targeted tandem dimer Tomato (mT). Upon Cre recombinase activation, mT is excised and membrane-targeted green fluorescent protein (mGFP) is expressed. In the *Sox2*^CreER^; *R26*^mT/mG^ mice, the Cre recombinase activity is triggered upon exposure to 4-Hydroxytamoxifen (4-OHT) solely in Sox2-expressing cells, leading to the expression of mGFP in these cells, as well as in their progeny. To induce *R26*^mT/mG^ recombination, 8-week-old *Sox2*^CreER^; *R26*^mT/mG^ mice were injected intraperitoneally with 4-OHT (H7904, Sigma-Aldrich, Buchs, Switzerland) dissolved in corn oil (100 mg/kg body weight). Twenty four hours after induction with 4-OHT, *Sox2*^CreER^; *R26*^mT/mG^ mice were sacrificed and processed as described below.

### Enzymatic Separation of Keratinized Epithelium From Mesenchyme of the Murine Tongue

**FIGURE 1:**
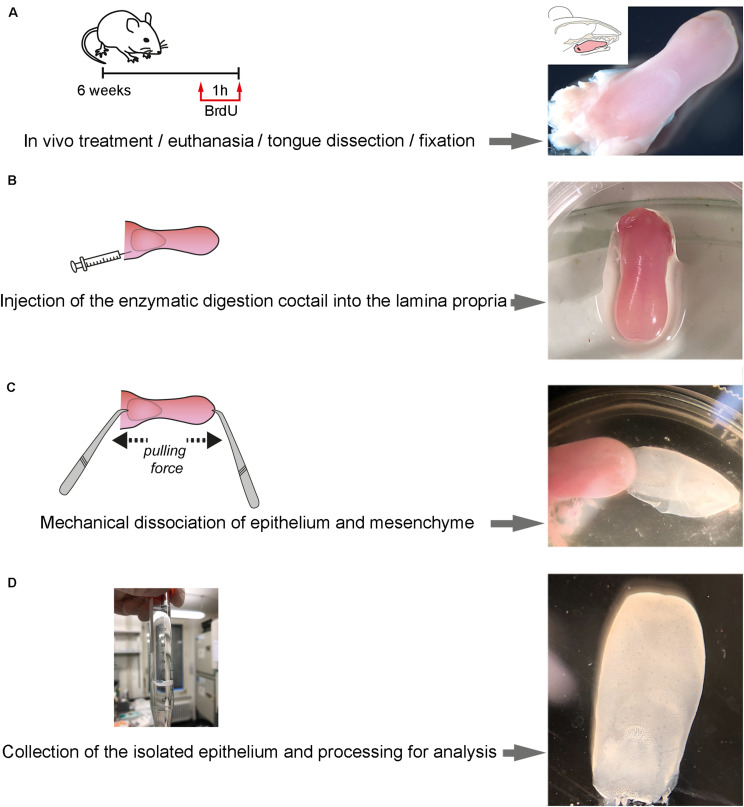
Step-wise procedure of enzymatic separation of tongue epithelium. **(A)** Schematic representation of the *in vivo* treatment and representative picture of the isolated tongue. A 6-week old mouse was injected with BrdU at 50 mg/kg. Upon 1 h of BrdU incorporation, the mouse was euthanatized and immediately after the tongue was dissected. **(B)** Schematic representation of the injection of the enzymatic cocktail at the lamina propria area of the tongue and representative picture of the treated isolated tongue. **(C)** Schematic representation of the procedure of mechanical separation of the tongue epithelium and representative picture. **(D)** Representative pictures of the separated epithelium at the end of the procedure.

All steps of the technique are shown in Figure 1.

Prepare a fresh solution of 2 mL of the enzymatic cocktail mixing collagenase A (at 1 mg/mL; Roche) and Dispase II (Sigma; D4693; at 2 mg/mL) in 0.1M PBS. Warm up to 37°C before usage.

1. Inject 50 mg/kg Bromodeoxyuridine (BrdU) in 0.9% NaCl solution (saline) via intraperitoneal injection and let the mouse rest for 1 h.
2. Sacrifice the mouse by CO_2_ overdose.
3. Position the mouse on its back and wash the facial region with 70% EtOH.
4. Open the mouth with tweezers, creating enough space to cut along the cheek pouch on both sides using scissors.
5. Remove the tongue by cutting along the lingual frenulum.
6. Wash the tongue with 0.1 M phosphate-buffered saline (PBS), pH 7.4, and transfer the tongue onto a Petri dish (Figure 1A).
7. Load a 1 mL syringe with a 27 G needle with 500 μL of the enzymatic cocktail.
8. Under the dissecting microscope, use a tweezer to hold the tongue in place and slowly insert the needle into the subepithelial space from the posterior cut end of the tongue (Figure 1B).
9. Move the needle deep into the tongue perforating the lamina propria, remaining parallel to the epithelial layer. Care should be taken not to puncture the epithelium. Inject the enzymatic cocktail while slowly retracting the needle.
10. Repeat step 8 for four spots in order to cover the maximum surface, injecting a total of 1 mL of enzymatic cocktail and allowing optimal distribution of the enzymatic cocktail throughout the subepithelial space of the tongue.
11. Transfer the tongue into a 1.5 mL Eppendorf tube, containing the same enzymatic cocktail and incubate the submerged tongue at 37°C for 30 min (5% CO_2_).
12. Transfer the tongue into a petri dish under the microscope. With one tweezer, hold the muscle of the tongue. With a second blunt tweezer, carefully grab the tip of the tongue, gently squeezing the epithelium. Slowly pull the muscle and epithelium in opposing directions (Figure 1C) (If resistance is encountered – possibly at the posterior cutting end – carefully lift the epithelium with the blunt tweezer.
13. Wash the separated epithelium with 0.1 M PBS, pH 7.4, and proceed to the desired application (Figure 1D).

### Keratin14 (K14), E-Cadherin, Green Fluorescence Protein (GFP), and Bromo Deoxyuridine (BrdU) Staining of Tongue Epithelium

Staining was performed as previously described (Mitsiadis et al., 2017; Jimenez-Rojo et al., 2019). Primary antibodies used: polyclonal rabbit anti-Keratin14 (dilution 1:100; Poly19053, Biolegend), polyclonal Goat anti-E-Cadherin (dilution 1:200, AF748, R&D Systems), rat anti-BrdU (dilution 1:50; OBT0300, AbD Serotec), polyclonal Rabbit anti-GFP (dilution 1:100; A11122; Invitrogen). The antibodies were incubated at 4°C overnight. Secondary antibodies used: Goat anti-Rabbit IgG 488, Alexa Fluor Plus 488 (dilution 1:1000; A32731, Invitrogen), Donkey anti-Goat IgG 568, Alexa Fluor Plus 568 (dilution 1:1000; Invitrogen); Goat anti-Rat 546, Alexa Fluor Plus 546 (dilution 1:1000; Invitrogen), Donkey anti-Rabbit IgG 488, Alexa Fluor 488 (dilution 1:1000; A21206: Invitrogen). These antibodies were incubated for 1 h at room temperature (RT).

### Confocal Imaging Analysis

All immunofluorescence images were produced using a Confocal Leica SP5 or SP8 with the following settings: 1,024 × 1,024 pixel dimension; 400 Hz laser; frame average 3 or 4. Photomultipliers PMT or Hyd. Fluorochromes used: Alexa 488, 546, and 594. A range of 25μm (standard) to 70 μm (whole mount) thick Z-stack was analyzed collecting images every 2.5 μm (standard) or 10 μm (whole mount). Images were processed using Imaris 8.0 software, Bitplane. Gaussian smoothing was applied to all channels when needed and equally to all images of the same batch.

### Processing of the Isolated Epithelium and Mesenchyme of the Tongue for RNA Isolation and cDNA Synthesis

1. After washing the separated epithelium and mesenchyme with 0.1 M PBS, transfer the tissues into 1.5 mL Eppendorf tubes and continue for RNA isolation or snap-freeze with liquid nitrogen for later proceeding.
2. Place the tissues on ice and add 500 μL of digestion buffer (RLT buffer; RNeasy Mini Kit, Qiagen) and homogenize the tissue on ice for 3 min using a motor driven grinder (Pellet Pestle Motor, Kontes).
3. Further isolation according to manufacturer’s instructions (RNeasy Mini Kit; Qiagen).
4. Quantification of the total RNA extracted by nanodrop.
5. RNA was retrotranscribed using the iScript^TM^cDNA synthesis kit (Bio-Rad Laboratories AG, Cressier FR, Switzerland) according to manufacturer’s instructions. Briefly, a template input of 1μg of total RNA was retrotranscribed into cDNA. Four microliter of 5x iScript reaction mix, 1 μl of iScript reverse transcriptase and 1 μg RNA was added per sample. In order to obtain a total volume of 20 μl, Nuclease-free water was added. The reaction mix was then incubated for 5 min at 25°C, for 30 min at 42°C and for 5 min at 85°C using a Biometra TPersonal Thermocycler (Biometra AG, Göttingen, Germany).

### Quantitative Real-Time PCR

1. The quantitative real-time PCR was performed using the CFX Connect Real-Time System and the software CFX Manager Version 3.1. Expression level analysis of β-*Actin* (housekeeping gene), *Vimentin*, *Smooth-Muscle Actin (SMA)*, *Keratin5, Keratin14*, and *P63* were performed using the SYBR^®^ Green PCR Master Mix (Applied Biosystems, Carlsbad CA, United States) in combination with corresponding oligonucleotide primers (Table 1).

**TABLE 1:** Murine primer pairs used for quantitative real time PCR.

Target	FW primer 5’–3’	RV primer 5’–3’
β-Actin	CATTGCTGACAGGAT GCAGAAGG	TGCTGGAAGGTGGAC AGTGAGG
P63	GGA AAA CAA TGC CCA GAC TC	GAT GGA GAG AGG GCA TCA AA
Keratin 5	GAA CAG AGG CTG AGT CCT GGT A	TCT CAG CCT CTG GAT CAT TCG G
Keratin 14	AGC GGC AAG AGT GAG ATT TCT	CCT CCA GGT TAT TCT CCA GGG
Vimentin	TGA AGC TGC TAA CTA CCA GG	TCT CTC AGG TTC AGG GAA GA
SMA	GGA TGC AGA AGG AGA TCA CA	AGC TTC GTC GTA TTC CTG TT

2. All amplifications were done in two technical duplicates of 3 biological replicates. Using the following thermocycling conditions:
3. 95°C for 10 min.
4. 40 cycles of 95°C for 15 s.
5. 60°C for 60 s.

Melt curve analysis was performed at 95°C for 15 s, 60°C for 5 s and 95°C for 30 s. Expression levels were calculated by the comparative ΔΔCt method (2^–ΔΔCt^ formula), after being normalized to the Ct-value of the β-*Actin* housekeeping gene. For statistical analysis a student *t*-test was applied to determine differential expression in biological triplicates (*n* = 3 mice analyzed).

Primers for qRT-PCR analysis are displayed in Table 1.

## Results

**FIGURE 2:**
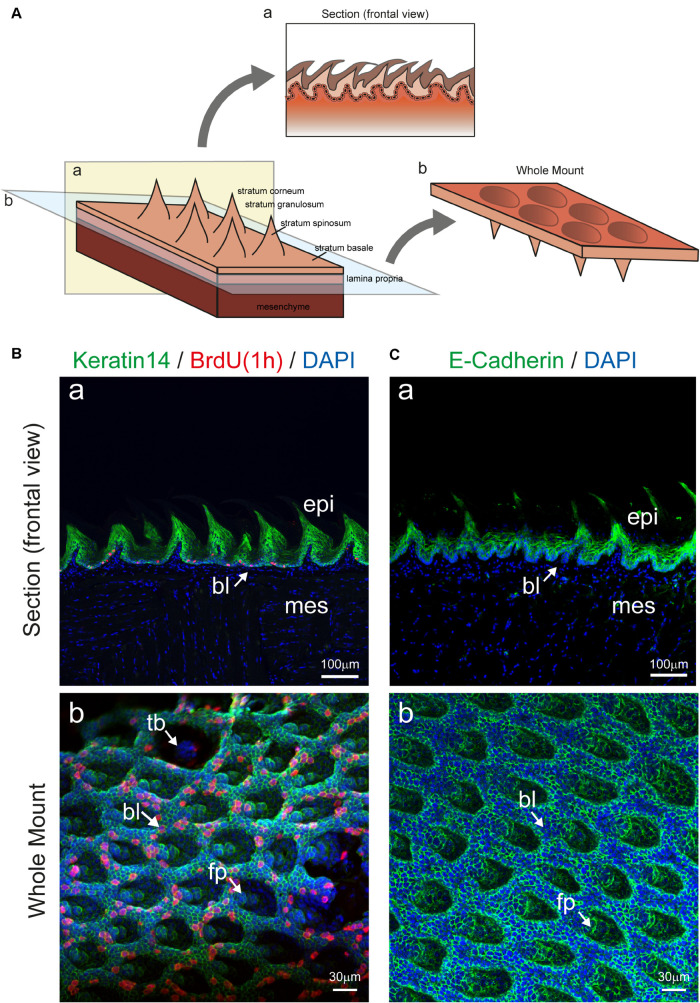
Imaging of tongue epithelial explant. **(A)** Schematic representation of the dorsal part of the tongue with epithelial and mesenchymal components. Panel a (yellow color) shows a section, displaying the mesenchyme, the lamina propria, and the epithelium with its different compartments. Plane b (blue color) depicts the location of mesenchyme and epithelial separation upon enzymatic digestion. The prospective rotation represents the exposed basal portion of the epithelium. **(B)** (a) Section of the tongue stained for Keratin14 (green), BrdU (red), and DAPI (blue). Arrow indicates the basal epithelial layer. (b) Whole mount immunofluorescent staining for Keratin 14, BrdU and DAPI of the isolated tongue epithelium. Arrows indicate important structures in the epithelium. **(C)** (a) Section of the tongue stained for E-Cadherin (green) and DAPI (blue). Arrow indicates the basal epithelial layer. (b) Whole mount immunofluorescent staining for E-cadherin and DAPI of the isolated tongue epithelium. Arrows indicate important structures in the epithelium. Abbreviations: bl, basal layer; epi, epithelium; fp, filiform papillae; mes, mesenchyme; tb, taste bud. Scale bars: Ba, Ca: 100 μm; Bb, Cb: 30 μm.

We show here that the enzymatic separation of the epithelium from the rest of the murine tongue does not alter the integrity and architecture of the epithelial tissue (Figures 1, 2). Advanced confocal imaging was used and we observed that the three-dimensional (3D) structure of the epithelium remained unaffected after the tissue separation procedure. We first compared frontal sections of embedded tissue stained in parallel with the whole-mount epithelial tissue (Figure 2) to precisely evaluate the structure of all epithelial layers. Proliferating progenitors (BrdU^+^) were located in the basal epithelial layer, while the staining was absent from cells composing the other layers of the epithelium (Figure 2Ba). Staining for Keratin14 was also detected in the various epithelial cell layers of the tongue (Figure 2Ba). To confirm these findings, we performed whole-mount staining against E-Cadherin, which was confined to keratinocytes, allowing the visualization of the inner organization of the filiform papillae of the tongue (Figures 2Ca,b). Similarly, using confocal imaging on whole mount epithelium, we identified an intact basal layer containing proliferating cells (BrdU^+^) (Figure 2Bb).

**FIGURE 3:**
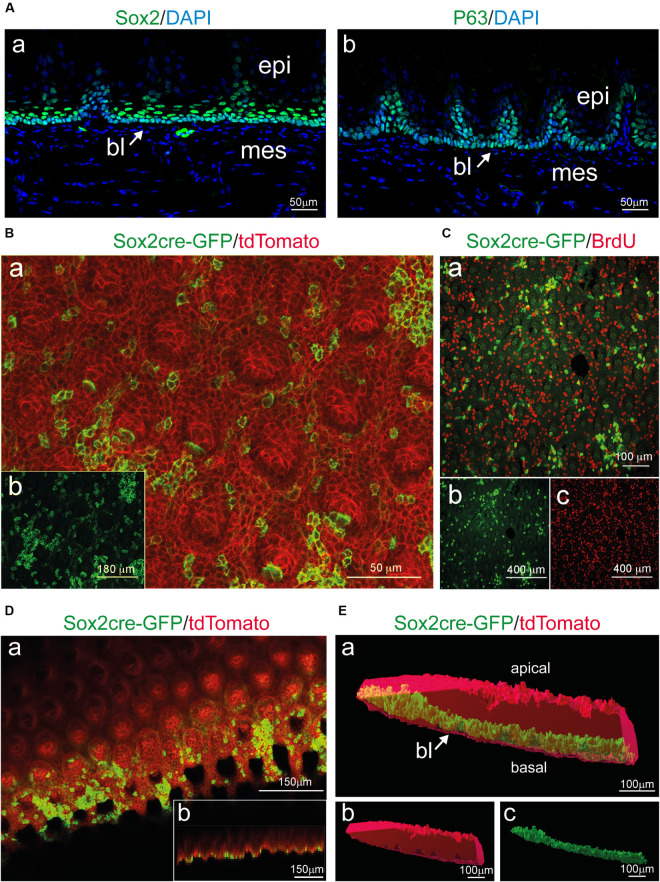
*Sox2* expression in the basal layer and whole mount imaging of tongue epithelial explants from induced *Sox2*^CreER^; *R26*^mT/mG^ mice. **(A)** Cross-section of the tongue stained for (a) Sox2 and (b) P63 identify progenitor cells in the basal layer (bl) of the epithelium (epi). Mesenchyme (mes) appears negative for both stainings. Arrows indicate the basal epithelial layer. **(B)** (a) Whole mount immunofluorescent staining for Sox2cre-GFP (green) and tdTomato (red) of the isolated tongue epithelium. (b) Single-channel image showing Sox2cre-GFP immunofluorescent staining (green color). **(C)** (a) Whole mount immunofluorescent staining for Sox2cre-GFP (green) and BrdU (red) of the isolated tongue epithelium. (b) Single-channel image showing Sox2cre-GFP immunofluorescent staining. (c) Single-channel image showing BrdU immunofluorescent staining. **(D)** (a) Whole mount immunofluorescent staining for Sox2cre-GFP (green) and tdTomato (red) of the isolated tongue epithelium displaying x-y orientation of the tissue. (b) Lower panel shows projection of the z-x axis **(E)** (a) 3D surface rendering of isolated epithelial tissue (red) with surface rendering displaying *Sox2*^+^ cells (green) in the basal layer of the isolated tissue. Arrow indicates the basal epithelial layer. (b) Single channel 3D surface rendering (red) of isolated epithelial tissue. (c) Single channel 3D rendering displaying *Sox2*^+^ cells (green) in the basal layer of the isolated tissue. bl, basal layer; epi, epithelium; mes, mesenchyme. Scale bars: Aa, Ab: 50 μm; Ba: 50 μm; Bb: 180 μm; Ca: 100 μm; Cb, Cc: 400 μm; Da, Db: 150 μm; Ea, Eb, Ec: 100 μm.

Enzymatic treatments used for the dissociation of the epithelial from the mesenchymal part of any tissue or organ act exclusively at the level of the basement membrane, which separates the two components and is composed by specific extracellular matrix molecules such as Collagen type IV and Laminin (Mitsiadis et al., 1995; Venkatesan et al., 2016). However, to confirm that the basal layer remained intact upon enzymatic dissociation, we analyzed the expression of previously reported basal epithelial markers (Okubo et al., 2009; Arnold et al., 2011; Ohmoto et al., 2017). We first compared the distribution of Sox2 and P63 proteins in cryo-sections to identify the basal and suprabasal epithelial layers (Figures 3A,B). We then used *Sox2*^CreER^; *R26*^*rmmT/mG*^ transgenic mice for further analysis of *Sox2*^+^ cells in whole mount settings. Expression of GFP under the Sox2 promoter was induced in adult mice (8 weeks old) upon 4-OHT injection and monitored 24 h later, thus allowing the identification of *Sox2*-expressing cells at the moment of analysis. Thereafter, the separated epithelium was imaged by confocal microscopy as whole-mount and revealed the presence of GFP^+^ cells (green color; Figures 3B–E), which are the *Sox2*-expressing cells, thus strongly suggesting that the basal epithelial layer of the tongue stays intact after the enzymatic separation procedure. *Sox2*-expressing cells were rarely linked to proliferative events as indicated by BrdU staining (Figure 3C, BrdU-positive cells in red). At the same time, the rest of the tongue epithelium could also be used for confocal imaging as it retains the Tomato fluorescence. Therefore, using this system, we were able to image the *Sox2*-expressing (GFP^+^) cell population in the basal layer and the general structure of the epithelium (Tomato^+^) without applying additional cell-specific markers. Note that the analysis of circumvallate as well as foliate papillae structures is also possible, however, we focused on filiform papillae in our exemplifying imaging (Figure 2). We could therefore visualize the entire epithelial structure in its 3D architecture, with minimal manipulation from the experimenter, suggesting that all portions of the epithelium were maintained intact following the enzymatic separation procedures.

**FIGURE 4:**
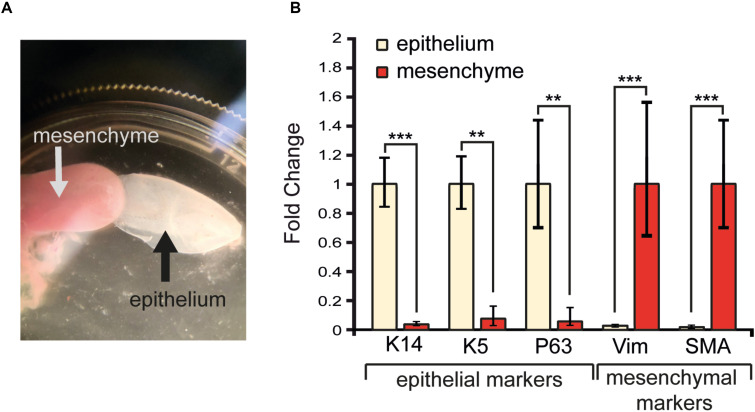
RNA extraction and analysis of transcripts in isolated tongue epithelium and mesenchyme. **(A)** Isolated mesenchyme and epithelium of the tongue upon enzymatic separation. **(B)** Gene expression levels of epithelial (*Keratin14*, *Keratin5*, *P63*) and mesenchymal (*Vimentin, SMA*) molecular markers upon tissue isolation. ^∗∗^*p* ≤ 0.01; ^∗∗∗^*p* ≤ 0.001.

To precisely characterize the degree of purity of the enzymatically-separated tissues and exclude any possibility of cross-contamination, we extracted the RNA from both epithelial and mesenchymal components. We analyzed several mesenchymal and epithelial transcripts, which encode for specific epithelial and mesenchymal proteins that are used as classical tissue-specific markers (Figure 4). P63 and Keratin5 are markers for epithelial progenitors residing in the basal layer of the tongue, while Keratin14 is expressed in all epithelial layers. The mRNA of *P63*, *Keratin14*, and *Keratin5* were highly expressed in the isolated epithelial tissue and only traces of these transcripts could be found in the mesenchymal portion of the tongue (Figure 4B). α-SMA and Vimentin are markers for mesenchymal cells: the α-SMA protein characterizes the smooth muscles of the tongue, while Vimentin is a major cytoskeleton component of mesenchymal cells (type III intermediate filament) (Fuchs and Weber, 1994; Ding et al., 2014). Transcripts for both *Vimentin* and *SMA* were highly expressed in the isolated mesenchymal compartment of the tongue, in contrast to the epithelial part where their expression was very low (Figure 4B). Taken together these results confirm the efficiency of the enzymatic separation method with the epithelial and mesenchymal tissue showing their specific genetic signature.

## Discussion

The tongue is a complex organ whose architecture and function depends on the proper organization of its various components. The tongue epithelium and mesenchyme are tightly interconnected. Enzymatic tissue separation procedures have previously been applied in regenerative approaches using 3D tongue epithelium and taste bud organoids derived from isolated epithelial stem cells (Ren et al., 2014; Aihara et al., 2015; Hisha et al., 2016; Hisha and Ueno, 2016; Palechor-Ceron et al., 2019). Several studies reported a variety of enzymatic cocktails for tongue tissue separation such as Pronase E/Elastase and Collagenase/Dispase II (Ookura et al., 2002; Ozdener et al., 2006; Venkatesan et al., 2016). Analysis of the 3D architecture of the detached tongue epithelium was accomplished using scanning electron microscopy (Nagato et al., 1989). Hydrochloric acid and tissue shaking were used for the isolation of the epithelium from the connective tissue of the tongue, which is not a representative method of the actual tissue dissociation protocols (Nagato et al., 1989; Venkatesan et al., 2016). Furthermore, the study used small epithelial square samples (2 mm) dissected from various parts of the rat tongue and not the complete tongue epithelium (Nagato et al., 1989). Advanced confocal imaging instead, allows the identification of intercellular and intracellular markers for a deeper molecular and functional analysis. The method described here was developed for optimal application in confocal imaging and RNA analysis. The applied technique requires a very short period of enzymatic treatment, resulting in the lowest possible rate of RNA degradation, and therefore it is the most appropriate methodology for downstream transcriptomic analyses. Another advantage is the fact that the whole tongue epithelium stays intact upon treatment, thus allowing the study of the different epithelial areas and structures of the tongue, such as the circumvallate and filiform papillae. Finally, this method requires minimal manipulation, which allows the visualization and processing of the tongue epithelium, closely representing its physiological state.

Previous studies have been abundantly based on single cell dissociation that *per se* alters cellular interconnections and does not allow the analysis of complex 3D tissues (Sukumaran et al., 2017). Cell-to-cell communications are a central element in the study of epithelial physiology, as they are strictly linked with epithelial function. Therefore, alteration of the anchoring molecules between cells should be prevented in order to keep structural integrity of the tongue and to enable studies on tissue physiology and/or pathology. Moreover, incomplete tongue epithelium separation from the mesenchymal tissue might result in sample contamination and lack of purity, hindering further biological analyses.

Since the tongue is exposed to continuous mechanical stress, the dorsal epithelium undergoes a continuous turnover, a process ensured by stem cells residing in the basal epithelial layer (Okubo et al., 2009; Tanaka et al., 2013). Sox2 is a master regulator of stemness in normal and pathological tissues (Sarkar and Hochedlinger, 2013; Feng and Wen, 2015). Recent studies in the tongue have shown that the conditional deletion of *Sox2* blocked the differentiation of both taste and non-taste epithelial cells, while the number of progenitor cells increased (Castillo-Azofeifa et al., 2018). Similarly, another study in tongue carcinoma demonstrated that SOX2 is a key regulator of the cancer epithelial cell plasticity (Yao et al., 2019). Our results confirm that Sox2 expressing cells and their progeny are located in the basal layer of the detached tongue epithelium, as previously described (Figure 3; Suzuki, 2008). Similarly, proliferating progenitors (BrdU^+^) were confined to the basal epithelial layer, and the distribution of both Keratin14 and E-Cadherin proteins was unchanged in all cell layers of the isolated epithelium (Figure 2). Taken together, these findings indicate that the 3D architecture of the tongue epithelium is maintained, and its characteristic cellular and molecular signature is preserved upon the enzymatic tissue separation procedure. We observed a peculiar 3D structure inside the filiform papillae, with a cavity extending toward the keratinized portion of the tongue. Interestingly, the inner portion of the filiform papillae identified by whole mount imaging contains clusters of keratinocytes grouping together to form the core of the structure. Additional investigation using cell-specific markers might reveal the entity of this cellular organization. Since the entire lingual epithelium was obtained upon dissociation, studies focussing on developmental events at the posterior portion of the tongue epithelium (e.g., circumvallate and foliate papillae) could be realized.

Our qRT-PCR analysis revealed a very strong expression of *Keratin14*, *Keratin5* and *P63* in the epithelium, but not in the mesenchyme. Conversely, *Vimentin* and *SMA* transcripts are mainly found in the isolated mesenchymal compartment of the tongue (Figure 4). Therefore, this separation technique is ideal for the molecular and cellular analyses of the epithelial part of the tongue, as both imaging and RNA analysis reflect an appropriate isolation of the epithelium portion from the rest of the tongue.

Tongue squamous epithelial cell carcinomas are frequent oro-oesophageal pathologies with severe prognostic outcomes (Upadhyay et al., 2016; Sawangarun et al., 2018; Porcheri et al., 2019; Yao et al., 2019). The 5-year overall survival rate after conventional treatment is ∼40–50%, due to treatment resistance, recurrence of the tumor, and consequently lymph node metastasis (Sommers et al., 2017; Mitsiadis, 2019; Porcheri et al., 2019). Therefore, the applied tissue separation methodology, combined with 3D confocal reconstruction techniques, might also allow the study of important tongue epithelium alterations during tumorigenesis (e.g., immune cell infiltration, cell proliferation, gene and protein regulation, and stem cell behavior).

In conclusion, the present results demonstrate that the architecture of the tongue epithelium is preserved and its molecular hallmarks are maintained upon enzymatic tissue separation techniques. Therefore, further experimental studies aiming to analyze a variety of tongue-related physiological and pathological events could be confidently performed.

## Data Availability Statement

The raw data were generated at the University of Zurich. Derived data supporting the findings of this study are available from the corresponding author CP on request.

## Ethics Statement

All mice were maintained and handled according to the Swiss Animal Welfare Law and in compliance with the regulations of the Cantonal Veterinary Office, Zurich (Licence number: 151/2014).

## Author Contributions

All authors contributed to the conception and design of the work, the analysis and interpretation of data, drafting and revising the text of the manuscript, provided their approval for publication of the content, and agreed to be accountable for the content of the work.

## Conflict of Interest

The authors declare that the research was conducted in the absence of any commercial or financial relationships that could be construed as a potential conflict of interest.

## Abbreviations

4-OHT: 4-Hydroxytamoxifen
Bl: Basal layer
BrdU: Bromodeoxyuridine
BSA: Bovine serum albumin
cDNA: Complementary deoxyribonucleic acid
CO_2_: Carbon dioxide
DAPI: 4′,6-diamidino-2-phenylindole
ECM: Extracellular matrix
EDTA: Ethylenediaminetetraacetic acid
Epi: epithelium
EtOH: Ethanol
FBS: Fetal bovine serum
Fp: filiform papillae
HCl: Hydrogen chloride
Mes: mesenchyme
NaCl: Potassium chloride
PBS: Phosphate-buffered saline
PCR: Polymerase chain reaction
qrt-PCR: Quantitative real-time PCR
RNA: Ribonucleic acid
RT: Room temperature
SMA: Smooth muscle actin
Tb: taste bud.
